# The ubiquitin ligase RNF2 stabilizes ERα and modulates breast cancer progression

**DOI:** 10.1007/s13577-022-00810-5

**Published:** 2022-10-21

**Authors:** Lei Yuan, Xin Li, Huijie Yang, Huixiang Li

**Affiliations:** 1 School of Basic Medical Sciences, Zhengzhou University, Zhengzhou, 450001 People’s Republic of China; 2 Xinxiang Key Laboratory of Tumor Migration and Invasion Precision Medicine, Henan Collaborative Innovation Center of Molecular Diagnosis and Laboratory Medicine, School of Laboratory Medicine, Xinxiang Medical University, Xinxiang, 453003 Henan Province People’s Republic of China; 3 Department of Pathology, The First Affiliated Hospital of Zhengzhou University, Zhengzhou, 450001 People’s Republic of China

**Keywords:** RNF2, ERα, Breast cancer, Ubiquitination, Stabilize

## Abstract

Estrogen receptor α (ERα) is the most common clinical marker used for breast cancer prognosis and the classification of breast cancer subtypes. Clinically, patients with estrogen receptor-positive breast cancer can receive endocrine therapy. However, resistance to endocrine therapy has become an urgent clinical problem. A large number of previous studies have proven that posttranslational modification of the estrogen receptor is significantly related to endocrine therapy resistance. RNF2 is a member of the RING finger protein family that functions as an E3 ubiquitin ligase. Several studies have clarified that RNF2 is a critical regulator of ERα transcriptional regulation. In our current study, we identified RNF2 as an important posttranslational modification regulator of the estrogen receptor. RNF2 depletion inhibited breast cancer cell progression and ERα signaling activity. TCGA data analysis indicated that RNF2 was elevated in breast malignancies, while RNF2 depletion could drastically inhibit estrogen response gene expression on a whole-genome scale. TCGA data analysis revealed that RNF2 was positively correlated with ERα target gene expression. Further mechanistic studies showed that RNF2 was mainly localized in the nucleus and associated with ERα. The association increased ERα stability by inhibiting ERα K48-linked polyubiquitination. In conclusion, our study implicates nongenomic regulation by RNF2 on ERα protein stability and suggests that targeting RNF2 could be a promising strategy for breast cancer treatments.

**Supplementary Information:**

The online version contains supplementary material available at 10.1007/s13577-022-00810-5.

## Introduction

Breast cancer is the most common malignancy in women [1, 2]. According to the molecular pathological classifications, breast cancer can be divided into four groups: luminal A, luminal B, HER2-overexpressing, and triple-negative breast cancer [3]. Among them, the luminal A and B types of breast cancer are ERα positive, accounting for approximately 70% of breast cancers. Selective estrogen receptor modulators (SERMs), such as tamoxifen, can effectively control the progression of luminal-type breast cancers. Nevertheless, the development of endocrine resistance is the major challenge for breast cancer therapeutics [4, 5]. Several mechanisms have been confirmed or proposed for endocrine therapy, but the detailed mechanisms are still not clear [4].

The link between breast cancer carcinogenesis and estrogen signaling has been known for 40 years [6]. The transcriptional program of ERα is critical and important for breast cancer progression. ERα is a member of the ligand-dependent superfamily of nuclear receptors of transcription factors, and the activity of ERα is regulated by estrogens, including estradiol and estriol [7]. The ERα protein is composed of three functional domains, including the transcriptional activation domain (AF1), the DNA binding domain (DBD) and the ligand-binding domain (LBD). The LBD domain is responsible for interacting with estrogen and several transcriptional cofactors to control ERα signaling activity [8]. If ERα is activated by estrogen, the ERα protein can shuttle into the nucleus and bind to cis-regulatory DNA regions in the genome, which promotes the expression of ERα target gene such as TFF1 and GREB1 to enhance breast cancer progression [9].

In addition, regulation of ERα via numerous posttranslational modifications has been shown to play important roles in ERα signaling activity. For example, phosphorylation of the ERα protein at S305 and S537 sites can facilitate ERα transcriptional activity on its target genes [10]. Acetylation at several sites on the ERα protein can also induce conformational changes in ERα that subsequently affect ERα stability [11, 12]. In addition, recent studies have revealed that several atypical ubiquitination modifications on ERα can affect ERα stability and signaling activity. Our previous studies have shown that RNF31/RNF181 modulates ERα protein stability via monoubiquitination [13, 14]. Based on this, we hypothesize that multiple pathways of ubiquitination mediated by E3 ubiquitin ligases might not necessarily lead to proteasome-dependent degradation but rather might modulate ERα signaling activity.

RNF2 (RING1B, or ring finger protein 2) is a member of the RING finger family of proteins that is mainly located in the nucleus [15]. One of the most important findings is that RNF2 may be a component of the polycomb group, inducing the monoubiquitination of histone H2A at lysine 119 and regulating global gene expression and chromosome structure [16]. Interestingly, RNF2 has been reported to correlate with the occurrence and progression of several human malignancies [17–19], including breast cancer [20, 21]. In our study, we found that RNF2 is required for breast cancer progression and estrogen signaling activity and that RNF2 can modulate ERα signaling by controlling ERα protein ubiquitination and stability.

## Materials and methods

### Cell lines

The human breast cancer cell lines T47D and MCF-7 and the human embryonic kidney cell line HEK293T were obtained from the American Type Cell Culture Collection. All cell lines were maintained at 37 °C with 5% CO2, and the cells were digested and passaged every 2–3 days according to ATCC recommendations. T47D cells were cultured with RPMI-1640 (Gibco, Grand Island, New York, USA) supplemented with 2 mM L-glutamine (25030, Life Technologies), 10% FBS (HyClone Laboratories, Logan, Utah, USA) and 1% penicillin/streptomycin (Beyotime, China). MCF-7 and HEK293T cells were cultured with Dulbecco’s modified Eagle’s medium containing 4.5 g/L glucose and 4 mM l-glutamine (DMEM, 41965, Life Technologies) that was supplemented with 10% fetal bovine serum and 1% cycloheximide. All cell lines were certified and were authenticated via short tandem repeat (STR) profiling using the PowerPlex 21 system.

### Plasmids and siRNA transfection

For plasmid transfection, cells were inoculated the day before transfection according to the Lipofectamine 2000 (Invitrogen, Waltham, Massachusetts, USA) manufacturer's instructions. The Myc-RNF2 plasmid was acquired from Addgene. The Flag-ERα, HA-Ub, HA-K48, HA-K63 Ubi, HA-K48R and HA-K63R plasmids were obtained from Ting Zhuang [22]. For siRNA transfection, Lipofectamine RNAiMAX (Invitrogen 13778-075) was used for transfection when the cells had reached approximately 50% confluence. RNF2 silencing was performed in MCF-7 and T47D cells using small interfering RNA (siRNA, GenePharma, China). The target sequences for the human RNF2 small interfering RNA were as follows: control siRNA sequence, TCGGTACTCAACCGTTAAG; RNF2 siRNA-1, ACGGAACTCAACCATTAAG; and RNF2 siRNA-2, TGGATGGTGCTAGTGAAAT.

### Quantitative real-time PCR (qRT–PCR)

Total RNA was isolated from cells using TRIzol (Sigma) and reverse-transcribed into cDNA using a PrimeScriptTM First-Strand cDNA Synthesis Kit (Cat. # 6110A, TaKaRa). mRNA expression was detected by SYBR Green qPCR assay (Cat. # 639676, TaKaRa). GAPDH was used as a control. The primer sequences used in this research were as follows: RNF2 F: 5-CAAACGGAACTCAACCATTAAGC-3, R: 5-CCACTTCTAAGGGCTGTGATG-3′; GREB1 F: 5′-CGT GTG GTG ACT GGA GTA GC-3′, R: 5′-ACC TCT TCA AAG CGT GTC GT-3′; ER F: 5′-GCT ACG AAG TGG GAA TGA TGA AAG-3, R: 5′-TCT GGC GCT TGT GTT TCA AC-3′; PS2 F: 5′-TGG GCT TCA TGA GCT CCT TC-3′, R: 5′-TTC ATA GTG AGA GAT GGC CGG-3′; and GAPDH F: 5′-TCGACAGTCAGCCGC ATCTT-3′ and R: 5′-GAGTTAAAAGCAGCCCTG GTG-3′. The data were analyzed using the 2-ΔΔCt method with GAPDH serving as a standard gene for normalization.

RNA was extracted with TRIzol, and RNF2 and GAPDH mRNA expression was measured using.

### Western blot analysis

Cells were harvested and lysed with Western and IP lysis buffer (Beyotime, P0013J) with a protease inhibitor cocktail (Roche, Indianapolis, IN, USA). The proteins were separated by SDS-polyacrylamide gel electrophoresis (PAGE) and transferred to a nitrocellulose membrane (Millipore). The following antibodies were used: anti-RNF2 (#5694, Cell Signaling Technology); anti-HA (MMS-101R, COVANCE); anti-Myc (Ab9106, Abcam); anti-Flag (20543-1-AP, Proteintech); anti-ERα (D8H8, 8644, Cell Signaling Technology); anti-ERα (SC-56833, 1:200); and anti-β-actin (A5441, Sigma). The membranes were then washed with PBST three times and incubated with peroxidase-conjugated AffiniPure goat anti-mouse IgG-HRP (A0216, Beyotime) or goat anti-rabbit IgG-HRP (A0208, Beyotime) secondary antibodies. The fluorescence signals were visualized using a Bio-Rad ChemiDoc (USA).

### Immunohistochemistry

The streptavidin-peroxidase-biotin (SP) immunohistochemical method was used to measure RNF2 protein expression in 65 paraffin-embedded breast tissues. paraffin-embedded tissues were cut into 3–5 μm sections and baked at 60 °C for 1 h. And then were deparaffinized with xylenes and rehydrated. Sections were submerged into EDTA antigenic retrieval buffer and microwaved for antigenic retrieval, and cooled at RT for 30 min. Non-specific binding sites were blocked by incubating the slides with 10% normal serum for 1 h at room temperature. For primary antibodies, sections of tissues were incubated with RNF2 (#5694, CST, 1:200) antibodies overnight at 4 °C. and then incubated with biotinylated goat anti-rabbit IgG antibody for 1 h at RT. After washing, the sections were incubated by biotinylated anti-rabbit secondary antibody, and then with streptavidin–horseradish peroxidase complex. Stained with diaminobenzidine, and the sections were counterstained with hematoxylin. After DAB and counterstaining with hematoxylin, sections were imaged using a Nikon upright microscope. Immunoreactivity was measured as depicted in previous paper [23]. This usage of clinical samples was reviewed and approved by the Ethical Board at Zhengzhou University with written informed consent from all the patients.

### CCK-8 assay

Cell viability was measured via CCK-8 (C0038, Beyotime) analysis according to the manufacturer’s protocol. The human breast cancer cell lines T47D and MCF-7 transfected with siControl or siRNF2 were plated on 96-well plates at 3 × 10^3^ cells/well. The culture supernatant was removed, and fresh medium containing 10 μL of CCK-8 reagent (1:100) was added to each well. After another 1 h of cell culture at 37 °C, the absorbance was detected at 450 nm with a BioTeK ELx800 microplate reader (BioTeK, Winooski, Vermont, USA) at 0 h, 24 h, 48 h and 72 h. The measurement for every sample was conducted in triplicate.

### EdU assay

Cell proliferation was detected by EdU (5-ethynyl-20-deoxyuridine) assay using an EdU cell proliferation detection kit (RiboBio, R11078) according to the manufacturer's protocol. The human breast cancer cell lines T47D and MCF-7 transfected with siControl or siRNF2 were plated on 96-well plates at 1 × 10^4^ cells/well. The cell proliferation proportion was measured by ImageJ.

### Colony formation assays

The human breast cancer cell lines T47D and MCF-7 transfected with siControl or siRNF2 were harvested and pipetted well to generate a single-cell suspension in complete culture medium at a concentration of 1 × 10^6^/ml. The single-cell suspension was diluted to 2000 cells in every well of a 6-well plate. The cells continued to be cultured in the incubator at 37 °C with 5% CO2 for approximately 2 weeks, and then the colonies were stained with crystal violet (Beyotime, C0121). The cell colony formation proportion was measured by ImageJ.

### Dual-luciferase reporter assay

Luciferase activity was measured using a Dual-Luciferase Reporter Assay System (E1910, Promega, USA). The human breast cancer cell lines T47D and MCF-7 transfected with siRNF2 or siControl were seeded in 12-well plates. After the cells were approximately 70–80% confluent, 0.5 μg of the ERE luciferase reporter plasmid and 0.01 μg of Renilla plasmid were transfected using Lipofectamine Reagent 2000 (Invitrogen), and the luciferase activity was detected using a GloMax-Multi Jr (Promega-GloMax Promega, USA).

### Xenograft tumor model

Four-week-old female BALB/c nude mice were purchased from the Beijing Vital River Laboratory Animal Technology Co., Ltd. shControl or shRNF2 T47D cells were resuspended and injected into the right flank of each mouse (4 × 10^6^ cells/mouse) subcutaneously. The tumor sizes are measured every 7-day, tumor volume was measured and calculated using the following formula: Volume (mm3) = length × width ^2^/2. All animals were raised in a specific pathogen free (SPF) and free access to water and food with 12 h of light.

### Lentivirus transduction

For lentiviral transduction, the lentiviral shRNF2 vectors were generated into pLVX lentiviral vector using T4 DNA ligase (NEB, American). The sense strand of the nucleotide sequence encoding shRNA targeting RNF2 was 5-ACGGAACTCAACCATTAAG-3. The packaging of lentivirus was performed with 4 μg PLVX-shRNF2, 3 μg psPAX2 and 1 μg pMD2.G plasmid into HEK293T cells using Lipofectamine 2000, according to the manufacturer’s protocol. After 48 h, the culture supernatant was collected and filtered through a 0.45 μM filter. T47D cells in 6-well plates were transduced with 1 ml viral supernatant supplemented, 1 mL fresh 10% FBS DMEM with 8 μg/mL Polybrene (Solarbio, China). Stably transfected cells were cultured in the 10% FBS DMEM with puromycin 1 μg/ml (Beyotime, China).

### Immunofluorescence assay

MCF-7 cells were fixed with 4% paraformaldehyde at room temperature for 10–15 min, permeabilized with 0.25% Triton X-100 for 15 min and blocked with 3% BSA for 1 h at room temperature. The anti-RNF2 antibody (#5694, Cell Signaling Technology, 1:200) and mouse anti-ER α antibodies (SC-56833, 1:200) were used. The cells were then washed with PBS three times and incubated with Alexa Flour 647 (Invitrogen, USA) anti-rabbit and FITC-conjugated anti-mouse secondary antibodies (Invitrogen, USA). The nuclei were stained with DAPI (Sigma). Samples with only secondary antibodies and no primary antibodies were used as negative controls. Images were captured with a Nikon A+ laser scanning confocal system. The acquired pictures were further processed and assembled using ImageJ.

### Coimmunoprecipitation (Co-IP) assay

Total cell lysates were pre-cleared and incubated with 20 μl of Protein A + G Agarose (Beyotime, P2012) and rabbit IgG (Beyotime, A7016, 1:50) for 2 h at 4 °C, and immunoprecipitation was then performed with an anti-ERα antibody (D8H8, Cell Signaling Technology, 1:50) for 4 h at 4 °C. Rabbit IgG (Beyotime, A7016, 1:50) was used as the negative control. The bound protein was analyzed with anti-RNF2 (#5694, Cell Signaling Technology, 1:2000).

### Publicly available clinical data analysis

RNA-seq data (TCGA) on RNF2 in breast cancer were downloaded from the Gene Expression Omnibus (GEO) database (Assessing number: GSE137579). Expression in luminal, HER2-positive and triple-negative breast cancer tissues and normal tissues was analyzed with GraphPad Prism 8. Analysis of the correlation of RNF2 with ERα and ERα target genes (TFF1 and PDZK1) was carried out with data for 1080 breast cancer samples from the TCGA database. For gene set enrichment analysis (GSEA), HALLMARKS_ESTROGEN_RESPONSE_LATE gene sets were used and downloaded from the GSEA Molecular Signatures Database. GSEA was implemented using GSEA 4.1.0 software.

### Statistical analysis

Statistical analyses were carried out using GraphPad Prism 9 software. All the data are presented as the mean ± standard deviation for at least 3 independent experiments. The significance of differences was determined using two-tailed Student’s *t* test. A *P* value < 0.05 was considered to indicate statistical significance.

## Results

### RNF2 is required for breast cancer growth in vitro and in vivo

**Fig. 1:**
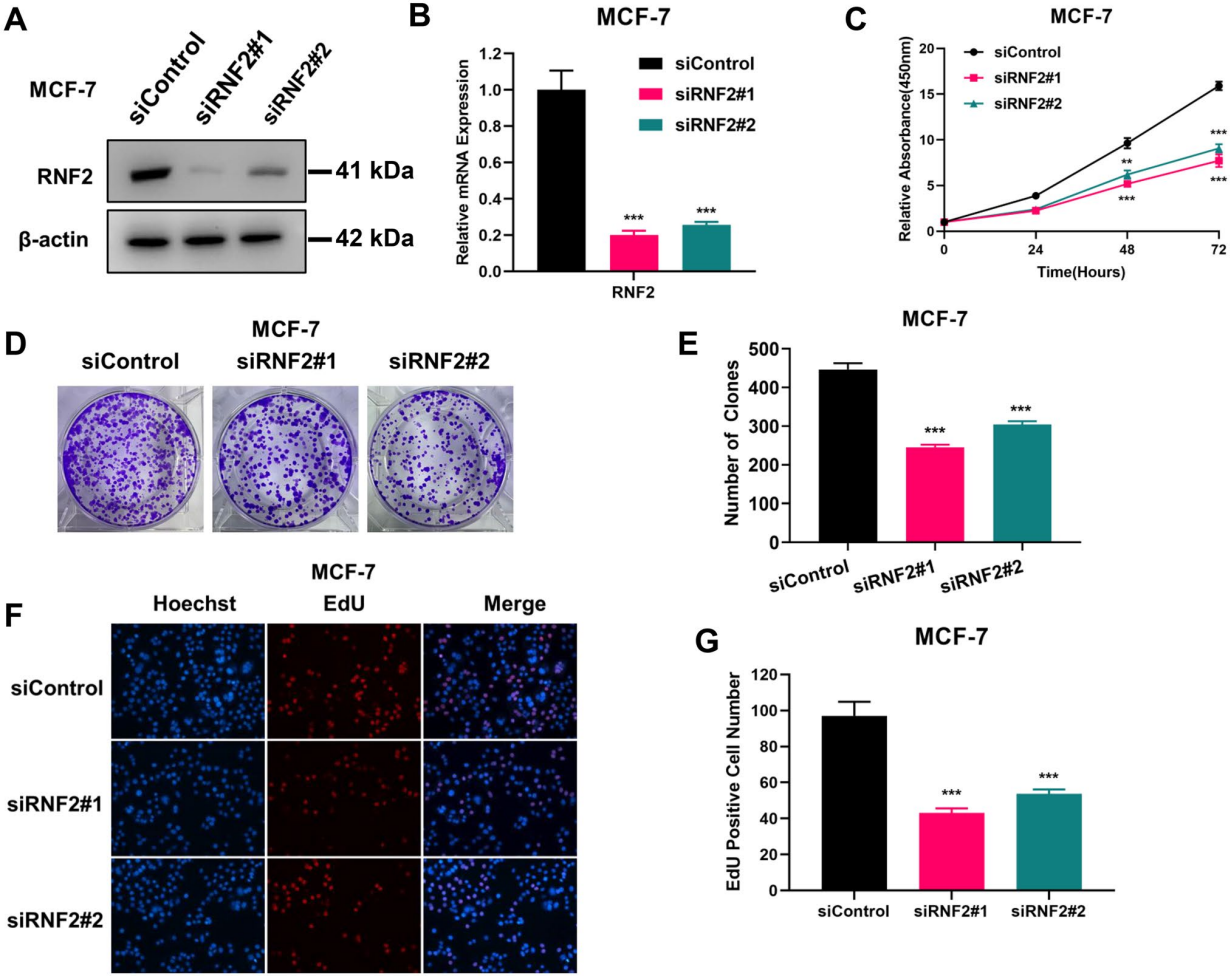
RNF2 knockdown inhibits proliferation in MCF-7 cells. **A** Western blot analysis of RNF2 expression in MCF-7 cells exposed to siControl or siRNF2. **B** mRNA expression levels of RNF2 in MCF-7 cells exposed to siControl or siRNF2. **C** Cell proliferation analysis was performed in MCF-7 cells transfected with siControl or siRNF2. **D** and **E** Cell growth was examined by colony formation assay in MCF-7 cells transfected with siControl or siRNF2. **F** and **G** Representative images of EdU assays in MCF-7 cells transfected with siControl or siRNF2. EdU-positive cells, red; cell nuclei, blue. The results are representative of 3 independent experiments. The data are the means ± SDs. **P < 0.01, ***P < 0.001 (Student’s *t* test)

We depleted RNF2 in MCF-7 cells to examine the effect on cell phenotype. RNF2 depletion was satisfactorily achieved, as validated via western blotting and qPCR (Fig. 1A-B). The CCK-8 assay showed that RNF2 depletion significantly inhibited cell growth in MCF-7 cells (Fig. 1C). The colony formation assay showed that RNF2 depletion inhibited the colony formation capacity of MCF7 cells (Fig. 1D, E),. In addition, we further carried out an EdU incorporation assay, which showed that RNF2 silencing reduced the number of EdU-positive cells (Fig. 1F, G).

The cell phenotype impact of RNF2 was further validated in another ERα-positive breast cancer cell line. We depleted RNF2 in T47D cells to examine the effect on cell phenotype. RNF2 depletion was satisfactorily achieved, as validated via western blotting and qPCR (Fig. S1A-B). The CCK-8 assay indicated that RNF2 depletion significantly inhibited cell growth in T47D cells (Fig. S1C). The colony formation assay indicated that RNF2 depletion inhibited the colony formation capacity in T47D cells (Fig. S1D-E). In addition, we further carried out an EdU incorporation assay, which indicated that RNF2 silencing reduced the number of EdU-positive cells (Fig. S1F-G).

**Fig. 2:**
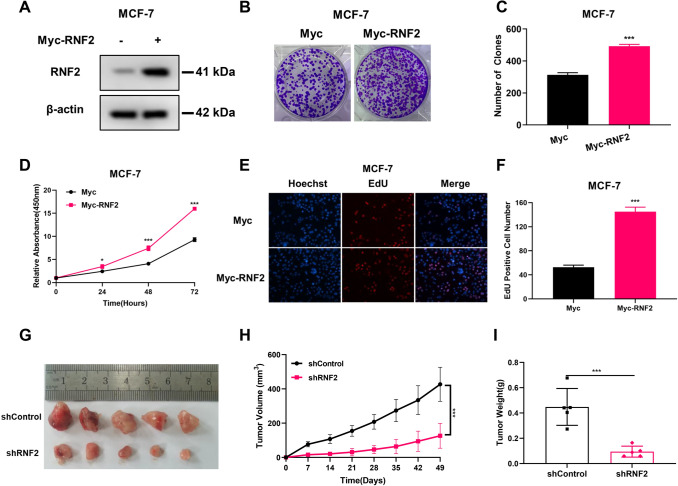
RNF2 is required for breast cancer growth in vitro and in vivo. **A** Western blotting analysis of RNF2 expression in MCF-7 cells exposed to Myc or Myc-RNF2. **B**, **C** Cell growth was examined by colony formation assay in MCF-7 cells transfected with Myc or Myc-RNF2. **D** Cell proliferation analysis was performed by CCK-8 in MCF-7 cells transfected with Myc or Myc-RNF2. **E**, **F** Representative images of EdU assays in MCF-7 cells transfected with Myc or Myc-RNF2. EdU-positive cells, red; cell nuclei, blue. **G**–**I** Representative images of tumors in nude mice subcutaneously inoculated with shControl or shRNF2 T47D cells (**G**). The tumor volume (**H**) and weight (**I**) in nude mice subcutaneously inoculated shControl or shRNF2 T47D cells. Results are representative of 3 independent experiments. Data are means ± s.d. *P < 0.05, **P < 0.01, ***P < 0.001 (student’s *t* test)

To further investigate the regulation of breast cancer cell phenotype by RNF2, we overexpressed RNF2 in MCF-7. The overexpression efficiency of RNF2 is shown in western blot (Fig. 2A). Colony formation assay showed that overexpression of RNF2 promoted the colony formation ability of MCF7 cells. (Fig. 2B, C). The CCK-8 assay showed that RNF2 overexpression significantly promoted cell growth in MCF-7 cells (Fig. 2D). In addition, we further carried out an EdU incorporation assay, which showed that RNF2 overexpression promoted the number of EdU-positive cells (Fig. 2E, F).

Finally, we further investigated the role of RNF2 in vivo through a xenograft mouse model. Our data showed that RNF2 silencing slowed tumor growth and reduced tumor weight in vivo. (Fig. 2G–I). These results demonstrate that RNF2 is required for breast cancer growth in vitro and in vivo.

### RNF2 expression is elevated in breast cancer and correlates with the expression of ERα target genes in human breast tumors

**Fig. 3:**
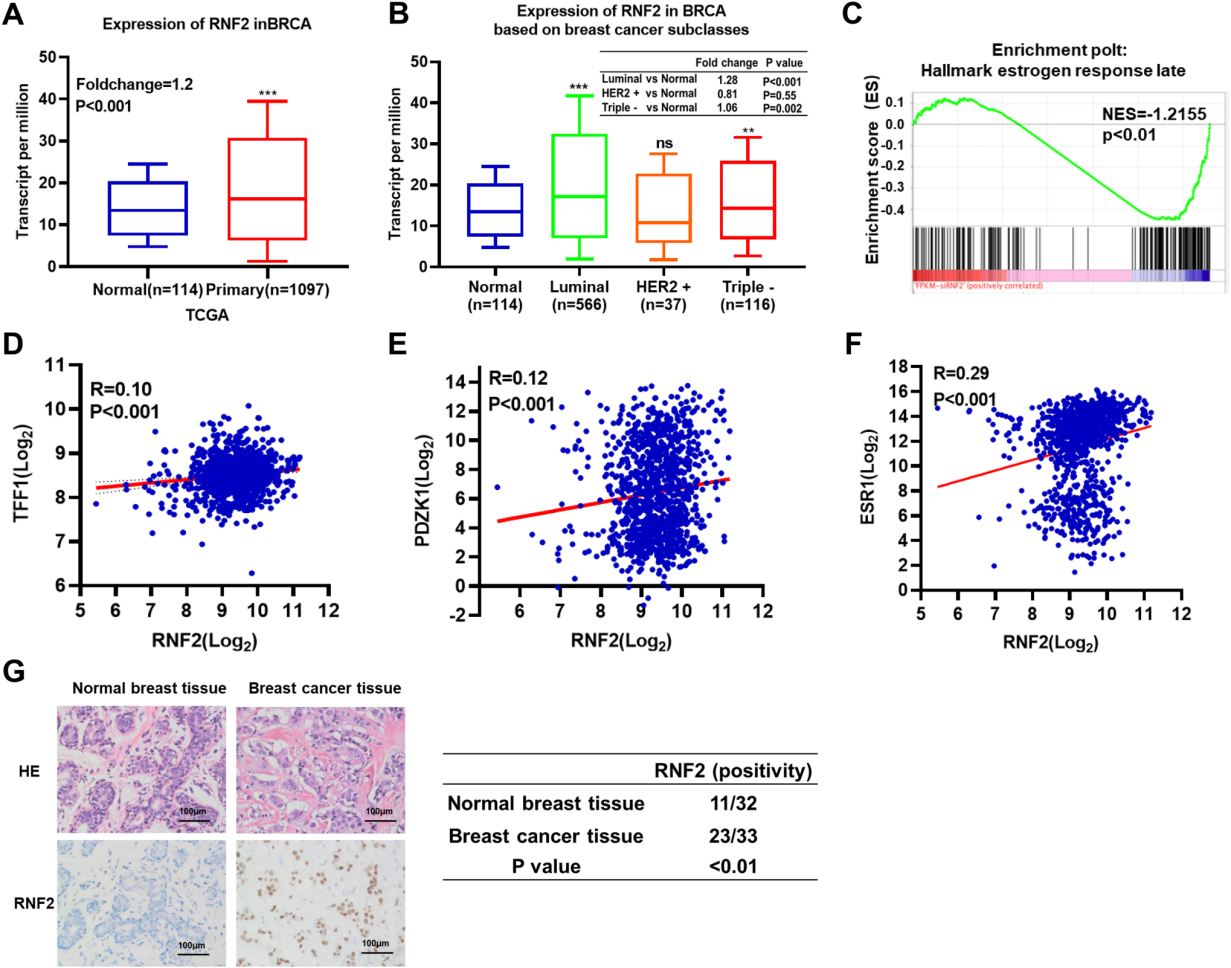
RNF2 is highly expressed in breast cancer and is associated with poor prognosis. **A** Expression distribution of RNF2 in primary cancer tissues and normal tissues using the TCGA breast database. ***P < 0.001 (Student’s *t* test). **B** mRNA expression of RNF2 in normal tissues and luminal, HER2-positive and triple-negative breast cancer tissues using the TCGA breast database. **P < 0.01, ***P < 0.001 (Student’s *t* test). **C** Gene set enrichment analysis (GSEA) showing enrichment of estrogen response genes in RNF2 shRNA T47D cells. **D**–**F** Publicly available data showing that RNF2 is positively correlated with ER α and the ER α target genes PDZK1 and TFF1 (https://www.cbioportal.org). **G** RNF2 expression is increased in human breast cancers compared with normal breast tissues immunohistochemistry analysis (11/32 vs 23/33; *P* < 0.01,)

To analyze the clinical impact of RNF2 in human breast tumors, we further investigated data from a publicly available database. Using data from the TCGA database, we observed that RNF2 mRNA levels were increased in breast tumors compared with normal breast tissues (Fig. 3A). The subclass analysis showed that RNF2 mRNA levels were elevated only in ERα-positive breast tumors (Fig. 3B). Further analysis of GEO data (GSE137579) from breast cancer cells showed that RNF2 depletion significantly inhibited estrogen response gene expression (Fig. 3C). TCGA database analysis of 1080 breast tumors showed that RNF2 expression was positively correlated with estrogen signaling target genes expression (PDZK1, TFF1 and ESR1, Fig. 3D–F). To investigate the expression of RNF2 at the level of breast cancer proteins, we analyzed RNF2 expression in breast cancer patient samples by immunohistochemistry (IHC). The expression of RNF2 was significantly increased in breast tumors compared to normal breast tissue (11/32 vs 23/33; *P* < 0.01, Fig. 3G). Besides, the pathological grade and lymph node metastasis data are also collected. The IHC analysis implicate that RNF2 expression correlates with ERα protein level, but no correlation with other with other molecular and clinical characteristics (Table 1). All these data show that RNF2 expression is positively correlated with estrogen signaling in clinical breast cancer samples.

**Table 1:**
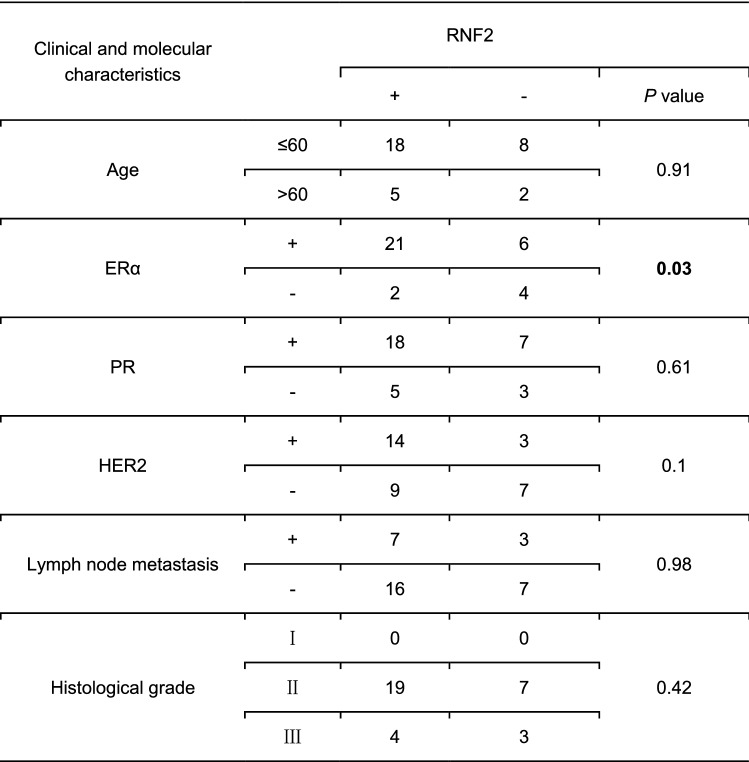
The correlation analysis between RNF2 expression and molecular/clinical characteristics in breast cancer samples

The bolded part shows that the *p *value < 0.05, which is statistically significant. RNF2 expression correlates with ERα in human breast cancer samples. The correlation analysis between RNF2 expression and molecular/clinical characteristics in breast cancer samples revealed

### RNF2 facilitates ERα signaling in breast cancer cells

**Fig. 4:**
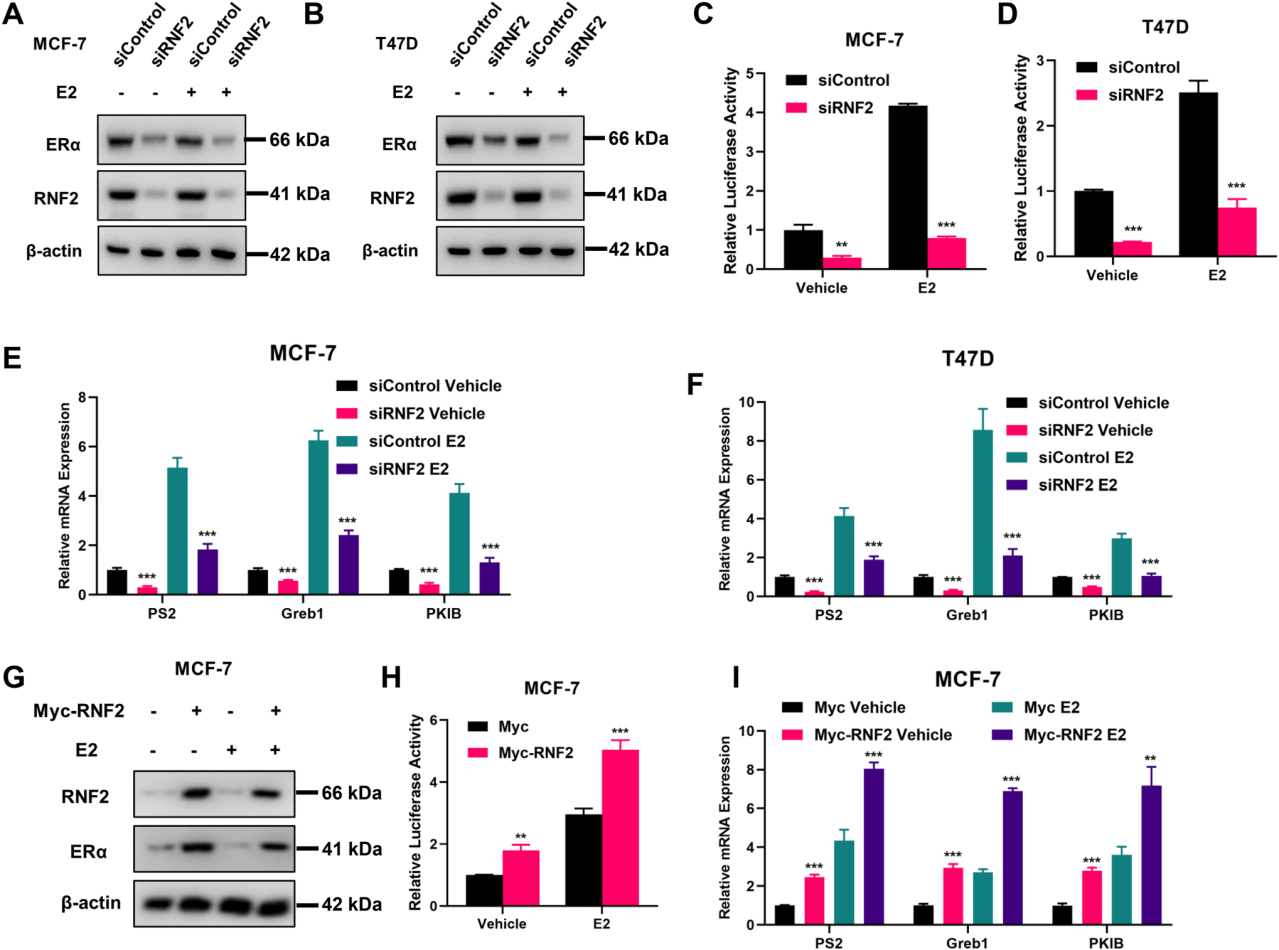
RNF2 silencing suppresses ERα signaling activity in breast cancer cells. **A** and **B** Western blot analysis of ERα and RNF2 expression in MCF-7 and T47D cells exposed to siControl or siRNF2. **C** and **D** Luciferase reporter assays in MCF-7 and T47D cells transfected with siControl or siRNF2. **E** and **F** Quantitative real-time PCR detection of the expression of the ER alpha target genes PS2, Greb1 and PKIB. MCF-7 and T47D cells were transfected with control or RNF2 siRNA for 48 h under hormone depletion conditions and then treated with 10 nM E2 or vehicle for 12 h. The results are representative of 3 independent experiments. The data are the means ± SDs. ***P < 0.001 (Student’s *t* test). **G** Western blotting analysis of ERα and RNF2 expression in MCF-7 cells exposed to Myc or Myc-RNF2 treated with 10 nM E2 or vehicle for 12 h. **H** Luciferase reporter assays in MCF-7 cells transfected with Myc or Myc-RNF2 treated with 10 nM E2 or vehicle for 12 h. **I** RT-qPCR detected ERα target genes PS2, GREB1 and PKIB expression. MCF-7 cells were transfected with Myc or Myc-RNF2 for 48 h treated with 10 nM E2 or vehicle for 12 h. Results are representative of 3 independent experiments. Data are means ± s.d. *P < 0.05, **P < 0.01, ***P < 0.001 (student’s *t* test)

We further investigated the effect of RNF2 on estrogen signaling in breast cancer cells. MCF-7 and T47D cells were used as the model cell lines. The immunoblotting data showed that RNF2 depletion significantly decreased ERα protein levels in both vehicle- and E2-treated conditions (Fig. 4A, B). The luciferase reporter assay indicated that RNF2 depletion inhibited estrogen response element activity in both vehicle- and E2-treated MCF-7 and T47D cells (Fig. 4C, D). We further examined classical ERα target gene expression in MCF-7 and T47D cells. Consistently, the qPCR data showed that RNF2 depletion inhibited the expression of ERα target genes, including PS2, GREB1 and PKIB, in MCF-7 and T47D cells (Fig. 4E, F).

To further investigate the regulation of estrogen receptor signaling pathway by RNF2, we overexpressed RNF2 in MCF-7. The immunoblotting data showed that overexpression of RNF2 promoted the expression of ERα proteins level in both vehicle- and E2-treated MCF-7 cells (Fig. 4G). We also investigated whether overexpression of RNF2 could affect the transcriptional function of ERα. We tested the luciferase activity of estrogen response element (ERE) in MCF-7 cells. The data showed that overexpression of RNF2 enhanced estrogen response element activity in both vehicle- and E2-treated MCF-7 cells (Fig. 4H). In addition, overexpression of RNF2 can also upregulate the expression level of ERα target genes, including PS2, GREB1 and PKIB, in both vehicle- and E2-treated MCF-7 cells (Fig. 4I).

### RNF2 associates with ERα and regulates its stability

**Fig. 5:**
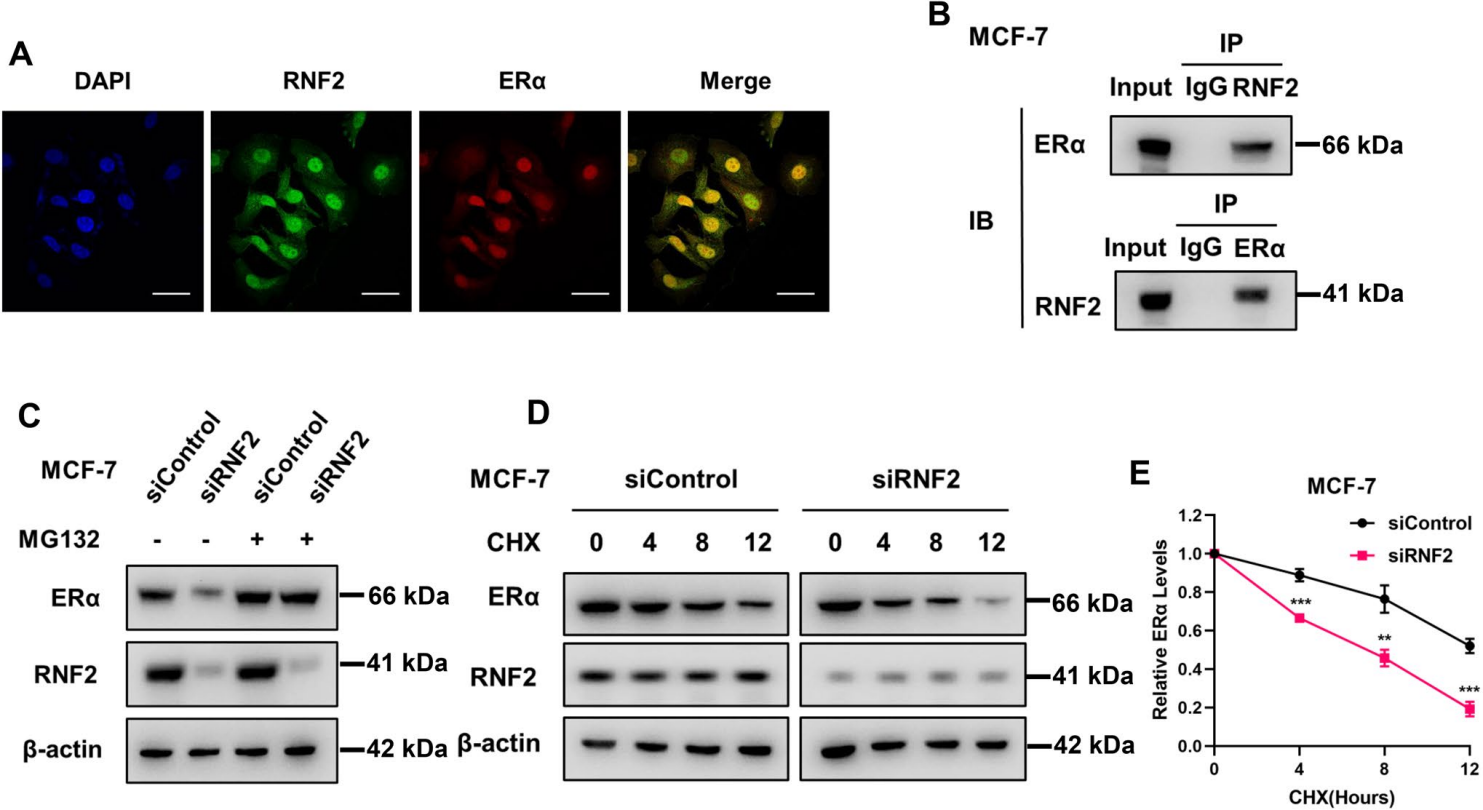
RNF2 binds to ERα and modulates ERα stability. **A** Immunofluorescence staining of ERα and RNF2 in MCF-7 cells; scale bar, 20 µm. **B** Coimmunoprecipitation experiments revealed that RNF2 can bind to ERα in MCF-7 cells. **C** RNF2 silencing decreased ERα protein levels, an effect that was diminished by MG132. MCF-7 cells were transfected with control or RNF2 siRNA for 48 h and then treated with MG132 (10 μmol/L) or vehicle for 8 h. **D** and **E** Western blot assays were used to detect the ERα protein half-life in MCF-7 cells. MCF-7 cells were transfected with siControl or RNF2 siRNA for 48 h and then treated with CHX (100 μmol/L) or vehicle for 8 h. The results are representative of 3 independent experiments

We further investigated the location of RNF2 and ERα in MCF-7 cells. The immunostaining data showed that RNF2 and ERα were mainly localized in the nucleus (Fig. 5A). The endogenous immunoprecipitation assay showed that RNF2 associated with ERα in MCF-7 cells (Fig. 5B). To further clarify whether the interaction of RNF2 and ERα is E2 dependent or not, we used endogenous immunoprecipitation assay in MCF-7 cell treated with vehicle or E2. The endogenous immunoprecipitation assay showed that RNF2 could interact with ERα is independent of E2 (Fig S2A). Subsequently, we wanted to investigate the biological impact of the interaction between RNF2 and ERα. We depleted RNF2 in MCF-7 cells and observed that RNF2 decreased ERα protein levels and that this effect was minimized in the presence of the proteasome inhibitor MG132 (Fig. 5C). In addition, RNF2 depletion coupled with cycloheximide treatment showed that RNF2 depletion significantly shortened the half-life of the ERα protein (Fig. 5D, E).

### RNF2 inhibits ERα K48-linked ubiquitination and degradation

**Fig. 6:**
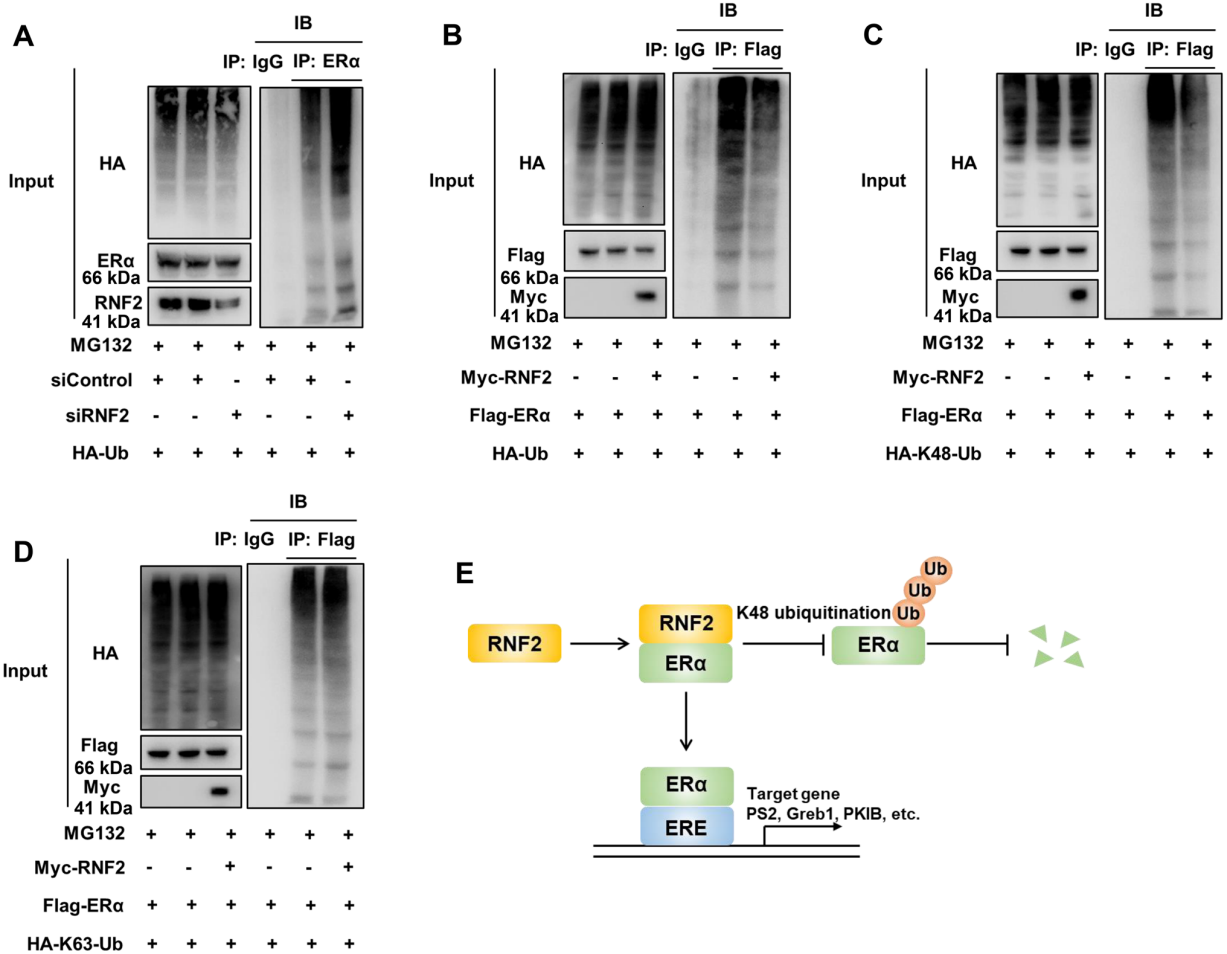
RNF2 facilitates ERα K48-linked ubiquitination. **A** Western blot analysis of polyubiquitinated ERα was performed after coimmunoprecipitation in MCF-7 cells. **B** Western blot analysis of polyubiquitinated ERα was performed after coimmunoprecipitation in HEK293T cells. **C** Western blot analysis of K48-specific polyubiquitinated ERα was performed after coimmunoprecipitation in HEK293T cells. **D** Western blot analysis of K63-specific polyubiquitinated ERα was performed after coimmunoprecipitation in HEK293T cells. **E** Schematic illustration of RNF2 associating with ERα and inhibiting ERα K48-linked ubiquitination and degradation in HEK293T cells. The results are representative of 3 independent experiments

Since RNF2 belongs to the family of RING E3 ubiquitin ligases, we investigated the effect of RNF2 on ERα ubiquitination. we validated RNF2 depletion increased the total polyubiquitination of ERα in MCF7 using endogenous proteins (Fig. 6A). The ubiquitination-based immunoprecipitation assay showed that RNF2 decreased the overall ubiquitination level of ERα in HEK293T cells (Fig. 6B). We further investigated the detailed ubiquitination mechanism affected by RNF2. We utilized K48-Ubi and K63-Ubi for further experiments. The data showed that RNF2 dramatically decreased K48-linked ubiquitination of ERα but had little effect on K63-linked ubiquitination (Fig. 6C, D). To further clarify the role of RNF2 in ER alpha stability in presence of E2, we performed ubiquitination-based immunoprecipitation assays in E2-treated conditions. The data showed that RNF2 could also dramatically decrease K48-linked ubiquitination of ER alpha in presence of E2 (Fig S3A). Our results demonstrate that RNF2 inhibits ERα K48-linked ubiquitination and degradation.

## Discussion

In the current study, we found that the RING finger E3 ubiquitin ligase RNF2 associates with the ERα protein in the nucleus in breast cancer cells, which subsequently facilitates breast cancer cell progression by stabilizing the ERα protein (Fig. 6E). Interestingly, RNF2 expression is elevated in breast cancer samples and correlates with ERα target gene expression. On this basis, targeting RNF2 to subsequently block its stabilizing effects on the ERα protein could be a plausible strategy for inhibiting breast cancer growth.

Based on the current knowledge about breast cancer, overactivation of ERα signaling is the major driver for carcinogenesis in 70% of breast cancer cases. Although several studies have confirmed that mutation or amplification of the ESR1 gene is not common in breast cancer samples, elevated expression of ERα could be more common in luminal breast tumors than in normal breast tissues [24]. Selective modulators of the ERα protein, such as tamoxifen, which effectively binds to the ligand-binding domain of ERα, could be effective therapies for breast cancer patients [25]. However, the development of endocrine resistance is still a clinical challenge for breast cancer therapy. Interestingly, most endocrine-resistant tumors maintain ERα expression, which could mean that ERα is still involved in the development of drug resistance. Several studies have reported that ERα can engage in crosstalk with several oncogenic pathways to transactivate signal transduction [26]. For example, ERα can associate with AKT signaling and facilitate breast cancer survival [27]. Based on this, modulation of ERα protein levels or expression is still an effective strategy for breast cancer treatment.

The ERα protein is subject to tight control by the ubiquitin–proteasome system in basal and ligand-induced conditions. Several ubiquitin ligases have been shown to induce ERα protein polyubiquitination and degradation, such as CHIP and MDM2. For example, the MDM2 protein can associate with ERα and facilitate ERα polyubiquitination and degradation [28]. However, recent studies have revealed several atypical ubiquitin ligases that can facilitate ERα monoubiquitination or K63-linked ubiquitination in a nonproteolytic ubiquitination manner. For example, RNF31 has been shown to associate with ERα and facilitate ERα signaling by inducing ERα monoubiquitination [8]. In addition, TRIM56 has been proven to modulate ERα signaling by inducing ERα K63-linked polyubiquitination [29]. In our study, we showed another E3 ubiquitin ligase, RNF2, to be a novel modulator in estrogen signaling.

Previous studies have shown that RNF2 belongs to the polycomb complex at monoubiquitination histone 3 [30], which functions as a transcriptional repressor. RNF2 has been found to be highly expressed in several human cancers and to be related to poor survival. Some studies have proposed that RNF2 can facilitate P53 degradation or suppress the formation of DNA replication forks. From this perspective, we propose that RNF2 might be an oncogene in human cancers. Furthermore, several studies have shown that RNF2 is closely related to the ER in breast cancer. Yusheng Zhang et al. reported that RING1B (RNF2) is a critical regulator of the estrogen receptor alpha (ERα) transcriptional regulatory circuit in ER+ breast cancer [21]. They proposed that RING1B promotes R-loop formation at ER target genes via direct participation in transcription of these genes, such as GREB1, and increased ER signaling [31]. However, Ho Lam Chan et al. showed that RNF2 is overexpressed in breast cancer and that it functionally associates with ERα and its pioneer factor FOXA1 in ER+ breast cancer cells by promoting the expression of oncogenes and/or regulating chromatin accessibility [32].

The findings of our current study result in conclusions similar to those of these two previous studies, namely, that RNF2 is elevated in ER+ breast cancer and promotes ER signaling target gene transcription. These findings further validate the conclusion that RNF2 mainly functions as an oncogene in breast cancer. However, our molecular assays reveal a different regulatory mechanism. In our study, RNF2 facilitated ERα signaling and promoted cell proliferation in breast cancer by enhancing ERα stability, possibly by inhibiting ERα K48-linked polyubiquitination. This interesting finding not only increases the understanding of ER alpha posttranslational modifications but also implies that RNF2 has multiple functions in different regulatory mechanisms for ERα.

In conclusion, our findings suggest that RNF2 is a novel modulatory component of ERα signaling in human breast cancer. Based on previous studies showing that RNF2 is an oncogene that promotes DNA replication and suppresses P53 function, targeting RNF2 might be a promising therapy for human cancers, especially luminal breast cancer.

## Supplementary Information

Below is the link to the electronic supplementary material.

Supplementary file1 Supplementary Fig. 1 RNF2 knockdown inhibits proliferation in T47D cells. (A) Western blot analysis of RNF2 expression in T47D cells exposed to siControl or siRNF2. (B) mRNA expression levels of RNF2 in T47D cells exposed to siControl or siRNF2. The results are representative of 3 independent experiments. The data are the means ± SDs. ***P < 0.001 (Student’s t test). (C) Cell proliferation analysis was performed in T47D cells transfected with siControl or siRNF2. (D and E) Cell growth was examined by colony formation assay in T47D cells transfected with siControl or siRNF2. The results are representative of 3 independent experiments. The data are the means ± SDs. ***P < 0.001 (Student’s t test). (F and G) Representative images of EdU assays in T47D cells transfected with siControl or siRNF2. EdU-positive cells, red; cell nuclei, blue. The results are representative of 3 independent experiments. The data are the means ± SDs. **P < 0.01, ***P < 0.001 (Student’s t test). Supplementary Fig. 2 (A) RNF2 and ER alpha endogenous immunoprecipitation assay in MCF-7 cell treated with 10 nM E2 or vehicle. Supplementary Fig. 3 (A) HEK293T cells were transfected with 2 µg of Flag-ER alpha plasmid, 0.5 µg of HA-K48 Ub plasmid, and 0.5 µg of Myc-tag or Myc-RNF2 plasmids. 24 h later, cells were treated with 10 nM E2 for 12 h. The cell extracts were immunoprecipitated with an anti-HA antibody. K48-specific polyubiquitinated ER alpha was detected via western blot analysis (PDF 267 KB)

## Abbreviations

ERα: Estrogen receptor α
RNF2: RING finger protein 2
AF1 domain: Activator function-1 domain
AF2 domain: Activator function-2 domain
DBD domain: DNA binding domain
PR: Progesterone receptor
HER2: Human growth factor receptor 2
ERE: Estrogen response element
ATCC: American type culture collection
STR: Short tandem repeat
IP: Immunoprecipitation
