# Supplementary figures and images for: The ubiquitin ligase RNF2 stabilizes ERα and modulates breast cancer progression
Source: Hum Cell. 2022 Oct 21;36(1):353–65. doi: 10.1007/s13577-022-00810-5 (PMC9813066; doi:10.1007/s13577-022-00810-5)

# Supplementary Figure 1

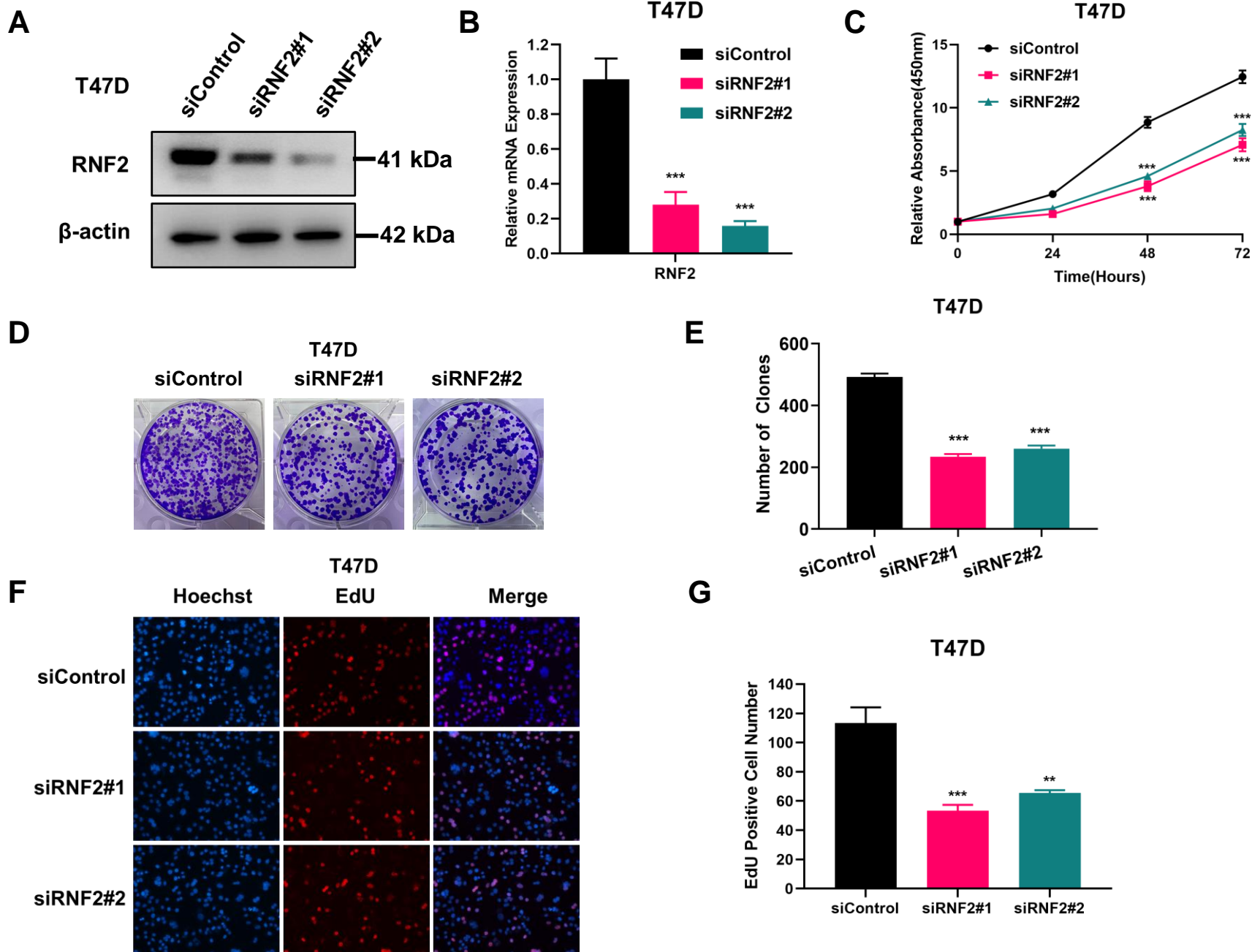

Supplementary Figure 2

A

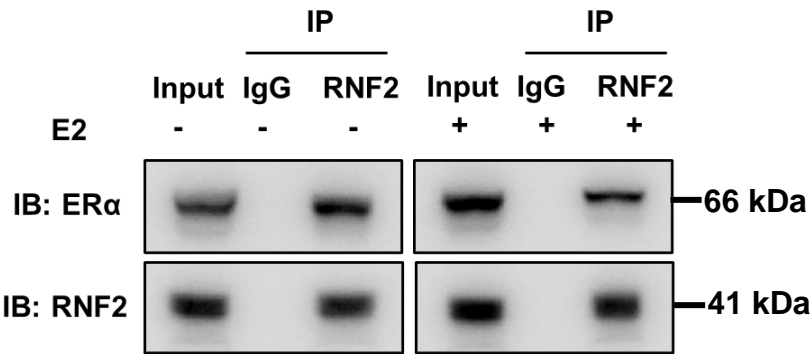

Supplement: Supplementary file 1 — Supplementary file1 Supplementary Fig. 1 RNF2 knockdown inhibits proliferation in T47D cells. (A) Western blot analysis of RNF2 expression in T47D cells exposed to siControl or siRNF2. (B) mRNA expression levels of RNF2 in T47D cells exposed to siControl or siRNF2. The results are representative of 3 independent experiments. The data are the means ± SDs. ***P < 0.001 (Student’s t test). (C) Cell proliferation analysis was performed in T47D cells transfected with siControl or siRNF2. (D and E) Cell growth was examined by colony formation assay in T47D cells transfected with siControl or siRNF2. The results are representative of 3 independent experiments. The data are the means ± SDs. ***P < 0.001 (Student’s t test). (F and G) Representative images of EdU assays in T47D cells transfected with siControl or siRNF2. EdU-positive cells, red; cell nuclei, blue. The results are representative of 3 independent experiments. The data are the means ± SDs. **P < 0.01, ***P < 0.001 (Student’s t test). Supplementary Fig. 2 (A) RNF2 and ER alpha endogenous immunoprecipitation assay in MCF-7 cell treated with 10 nM E2 or vehicle. Supplementary Fig. 3 (A) HEK293T cells were transfected with 2 µg of Flag-ER alpha plasmid, 0.5 µg of HA-K48 Ub plasmid, and 0.5 µg of Myc-tag or Myc-RNF2 plasmids. 24 h later, cells were treated with 10 nM E2 for 12 h. The cell extracts were immunoprecipitated with an anti-HA antibody. K48-specific polyubiquitinated ER alpha was detected via western blot analysis (PDF 267 KB) [file 13577_2022_810_MOESM1_ESM.pdf]
